# Learnings From an Innovative Model to Expand Access to a New and Underutilized Nonhormonal Contraceptive Diaphragm

**DOI:** 10.9745/GHSP-D-24-00215

**Published:** 2024-10-29

**Authors:** Maggie Kilbourne-Brook, Patricia S. Coffey

**Affiliations:** a PATH, Seattle, WA, USA.

## Abstract

We document the effort over the last 30 years to respond to the call by women advocates at the International Conference on Population and Development for more woman-initiated single or dual-purpose contraceptive methods by developing the Caya contoured diaphragm, an innovative diaphragm designed to meet the needs of women and their partners and expand options for nonhormonal barrier contraception. We describe the complex and interrelated set of activities undertaken to develop the product using a human-centered design process and how we are working to create a corollary sustainable market. This review includes the evidence generated around improved acceptability among couples in low- and middle-income countries and depicts challenges and practical actions on how to dispel misconceptions about diaphragm use. Importantly, we share programmatic lessons learned on increasing universal access to this new sexual and reproductive health technology. Following our new model for increasing access to new and underutilized methods, Caya is now registered and being marketed in nearly 40 countries worldwide.

## Abstract (precis)

Experiences with early introduction of the contoured Caya diaphragm provide a new model for increasing access to new and underutilized methods, especially through private-sector partners.

## Teaser

**Key Findings:**

- The International Conference on Population and Development call for research on female barrier methods led to the Caya contoured diaphragm, an innovative diaphragm designed to meet the needs of women and their partners and expand options for nonhormonal barrier contraception.
- This product contributes to strengthening universal access to sexual and reproductive health care by offering a woman-initiated family planning method particularly suited to those who want a nonhormonal method or have infrequent sex.
- Consumer experience is breaking down misconceptions about diaphragm uptake and acceptability across countries.
- Introduction through private-sector channels may pave the way for broader uptake and access.

**Key Implication:**

- Public-sector policymakers and program managers, as well as small business entrepreneurs, may use this summary of the product and market development of the Caya contoured diaphragm to spark national and regional dialogue about the most effective way to expand universal access to sexual and reproductive health by adding a novel nonhormonal barrier method to the contraceptive method mix offered in country.

## THE NEED FOR INNOVATIVE BARRIER CONTRACEPTIVE OPTIONS FOR WOMEN

The 1994 International Conference on Population and Development (ICPD) marked a paradigm shift for family planning (FP): moving away from top-down discussions on limiting population growth toward a recognition of reproductive health and its importance in improving the lives of women and couples.^1^^,^^2^ Around this same time, the Special Programme of Research, Development and Research Training in Human Reproduction (cosponsored by the United Nations Development Programme, United Nations Population Fund, UNICEF, World Health Organization, and World Bank) spearheaded dialogues that brought women’s health advocates into the process of defining research priorities through their Creating Common Ground meetings.^3^ These meetings brought women’s perspectives into discussions around contraceptive safety, efficacy, service delivery, and research on technologies in development.^4^^–^^6^ A unified voice from women reflected their collective call for more single or dual protection methods under their control, with few side effects, that could be used when needed.^3^^,^^7^

A unified voice from women reflected their collective call for more single or dual protection methods under their control, with few side effects, that could be used when needed.

In response to the ICPD call to action, PATH identified an existing underutilized woman-initiated barrier method—the contraceptive diaphragm—as being a promising option. Because diaphragms are recommended for use with a contraceptive gel and, at that time, multiple research groups were developing microbicide gel products that aimed to protect from both HIV and unintended pregnancy, it seemed likely that in the future, these products could be used together to help address women’s need for multipurpose protection. However, by the 1990s, diaphragm use in the United States had dropped from about 10% of contraceptive users in 1965 to about 3%, and the figure was even lower in other countries.^8^^,^^9^ Further, few studies had been conducted to evaluate use, acceptability, and fit parameters. Some stakeholders questioned the efficacy of this method, women’s ability to use it, and whether diaphragms were appropriate for women in low- and middle-income countries (LMICs) or resource-limited settings.

Some of this stakeholder bias was grounded in myths and misconceptions about diaphragms and diaphragm users. Because diaphragms were available primarily in high-income countries, some stakeholders believed only educated women from high-income countries could use diaphragms.^10^^,^^11^ Diaphragms were characterized as only used by women either who had infrequent sex, were in stable partnerships, or were nearing menopause.^12^ Other misconceptions included that diaphragms were difficult to use, especially by young women, because use requires comfort with vaginal touching, basic understanding of vaginal anatomy, and access to water and washing.^11^^,^^13^ It was also widely believed that women were unable to use barrier contraception effectively because it is user controlled. As hormonal contraception and intrauterine devices became more widely available and providers counseled on use of more highly effective methods, provider bias further stigmatized and reduced access.^12^ These perspectives are based on little evidence and discount women’s agency and ability to manage obstacles.

Women and their partners, providers, procurement agencies, and donors were engaged in early product development efforts to understand what made vaginal barrier methods difficult to use, supply, and provide. To deepen our understanding, we pioneered a user-centered process that put women’s needs at the center at a time when involving users as co-designers of reproductive health technologies was novel.^14^ These types of processes—broadly referred to as human-centered design and design thinking—are more common now and have been employed across a range of health and development areas to inform development of biomedical interventions for sexual and reproductive health and to improve health systems.^15^^–^^18^ Human-centered design is a set of tools and a problem-solving approach that overlaps with other strategies familiar to public health, such as sociobehavioral research, participatory research, implementation science, and patient-centered service delivery.^17^^,^^19^

## DESIGNING AND DEVELOPING A DIAPHRAGM RESPONSIVE TO WOMEN’S AND PROVIDERS’ NEEDS

Traditional diaphragms were supplied in up to 9 sizes, ranging from 55 mm to 95 mm in diameter, and had multiple spring styles. This made them difficult to stock and provide as clinics did not know how many of each size of diaphragm to order. Also, a clinic fitting was required to determine which size a woman could wear. The provider estimated the distance between the posterior fornix and the pubic bone in the vagina and fitted the largest-sized diaphragm a woman could comfortably wear, leading to a “wedged fit” protocol. The wedged fit contributed to discomfort for some women and pressure on the urethra.

Using a human-centered design process, PATH defined the performance objectives that a new diaphragm would be required to meet to address the needs and preferences of users and other key stakeholders (such as providers and donors) to address barriers that limited use of traditional diaphragms. The performance objectives guided development and were the benchmark against which prototypes were evaluated (Table 1), with the aim of building acceptability into the product along the way. Acceptability is characterized as the degree to which the product meets the needs and requirements of users and other stakeholders.

**TABLE 1.** Design Requirements for the Diaphragm Innovation Based on an Iterative Human-Centered Design Process

**Key Stakeholders**	**User/Stakeholder Perspectives on Traditional Diaphragms**	**Performance Objectives for Diaphragm Innovation**
Women	Difficult to handle during insertion. Difficult to remove. Uncomfortable to wear for 6 hours as recommended. Messy, slippery with contraceptive gel. Increased risk of urinary tract infection.	Easy to insert (especially for new users). Easy to remove. Comfortable for both partners. Less messy than traditional diaphragm.
Providers	Exam required to assess appropriate diaphragm size problematic. Not confident in the fit protocol. Perception that clients do not use diaphragms consistently. Perception of diaphragms as not effective; other methods are easier to provide and more effective.	Single-sized device eliminates need for pelvic exam and fitting. Streamline supply logistics.
Donor and procurement agencies	Multiple sizes make diaphragms logistically difficult to supply and provide.	Single-sized device fits most women (eliminates need for pelvic exam/fitting). Simplify device procurement.

The environmental impact of a new diaphragm also was considered as part of the development process due to interest in the impact of contraceptives and medical devices on the environment and waste stream.^20^ Because diaphragms are reusable for at least 2 years, they are more environmentally friendly than single-use barrier methods, such as male and female (internal) condoms. Also, the diaphragm is a nonhormonal method and, therefore, has fewer environmental and health impacts than hormonal methods. Additionally, a reusable diaphragm could be an environmentally friendly, low-cost way to deliver microbicide gel rather than using single-dose applicators for gel delivery (applicators were supplied in clinical studies).

Our multidisciplinary team included product development engineers, public health/sexual health experts, reproductive health researchers, and commercialization advisors. Employing a user-centered process, we implemented multiple iterative rounds of prototype evaluation and refinement to address issues that affected ease of use (especially for new users) and comfort for both partners. We explored various elements of the material, production process, and features that could lead to a refined design. PATH worked with partners in multiple countries to develop and validate an innovative diaphragm that would expand options for nonhormonal contraception.^14^ PATH used a similar process to develop a refined female (internal) condom design to address the needs of women and their partners regarding ease of use and comfort during sex.^21^

We evaluated prototype designs and features through iterative evaluations, first with women in clinic settings, then progressing to women evaluating the prototype at home, and eventually advancing to couples’ use studies among persons not at risk of pregnancy and not relying on the prototype for contraception. At-home evaluation of prototypes allowed women to practice insertion and removal and to wear the prototype when they were more relaxed and in control of their environment, as compared to being in a clinic setting.

**FIGURE 1:**
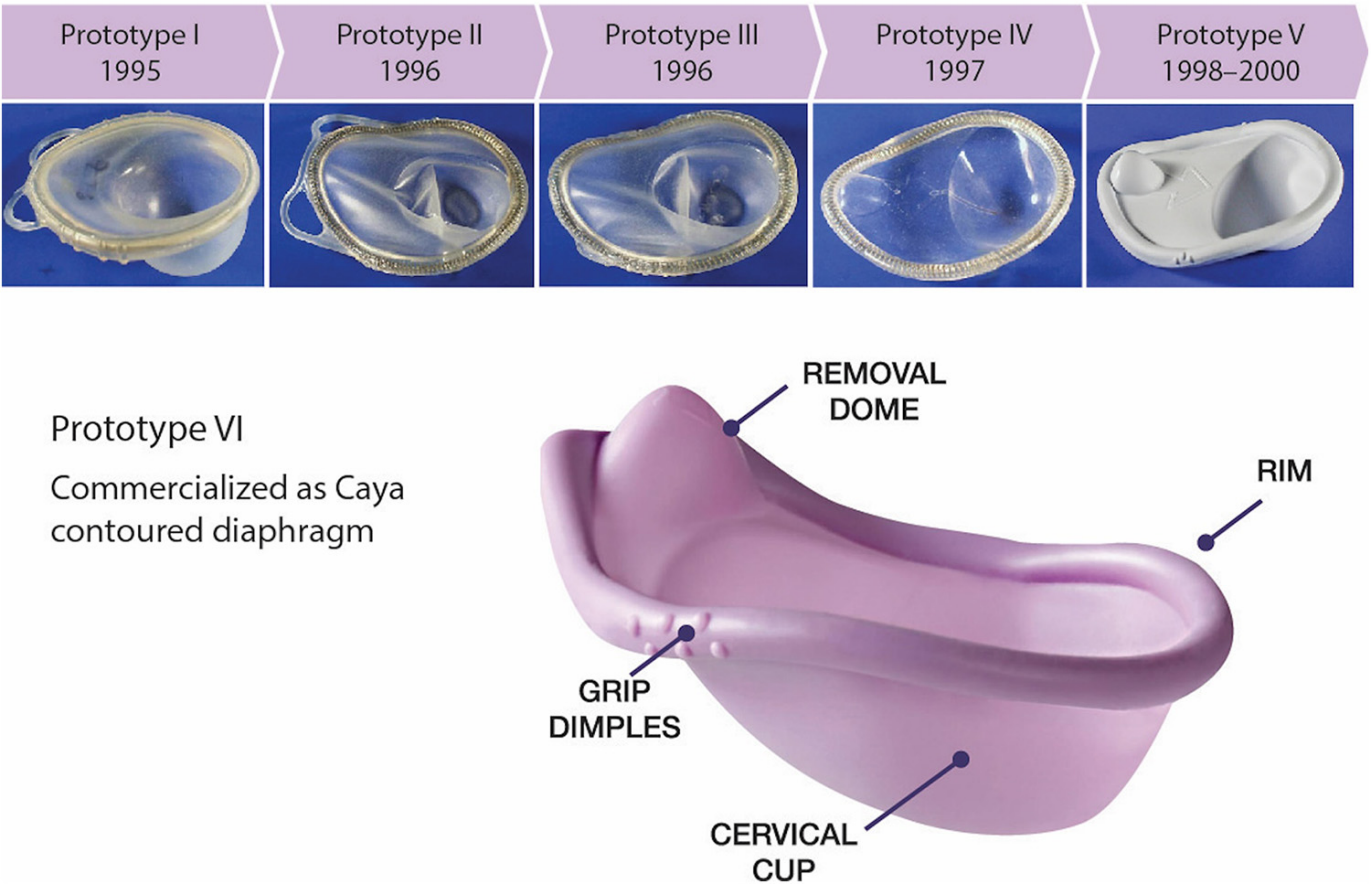
Six SILCS Prototype Iterations and the Final Design of the Caya Contoured Diaphragm

**FIGURE 2:**
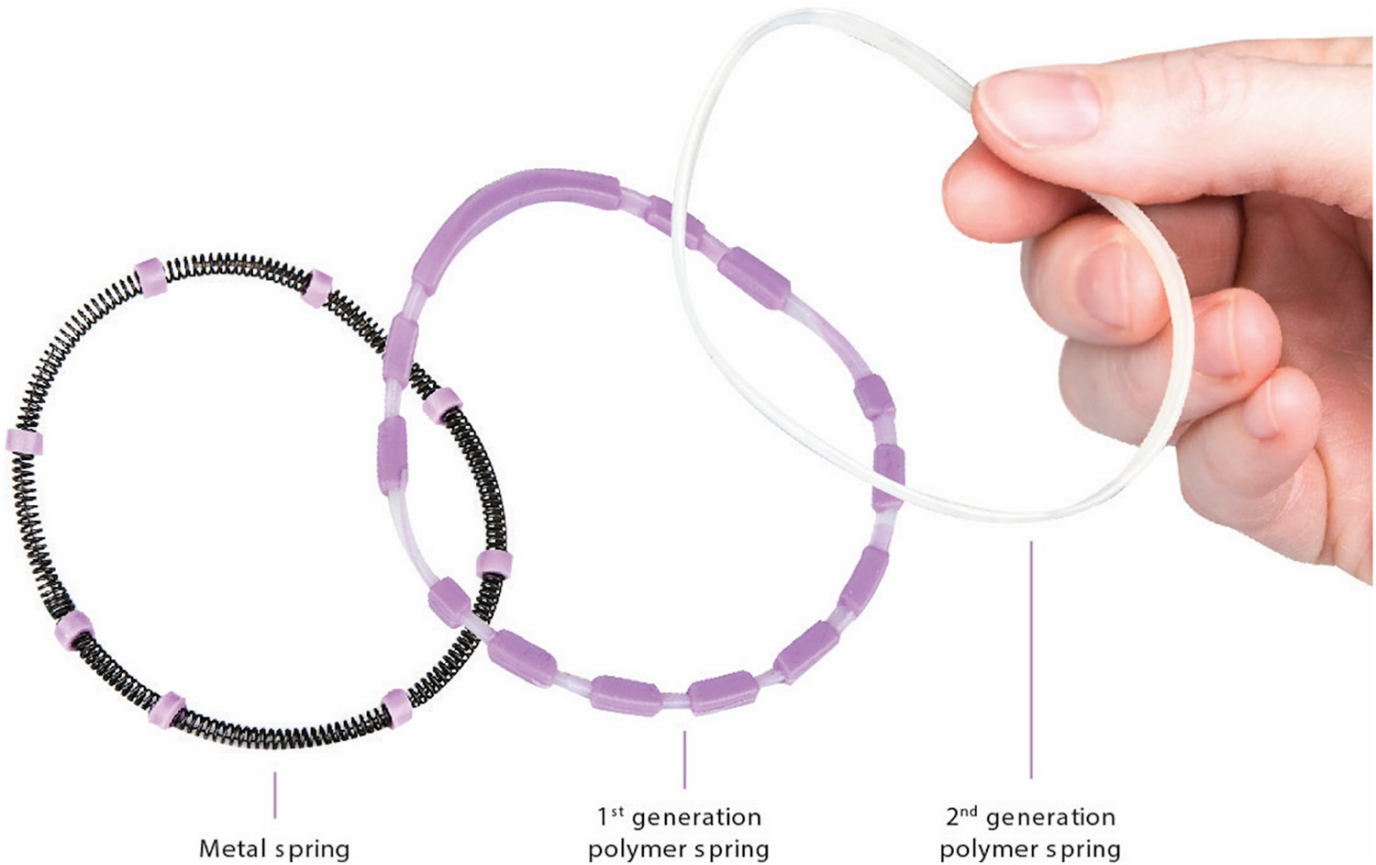
Contoured Spring of the Caya Contoured Diaphragm

**FIGURE 3:**
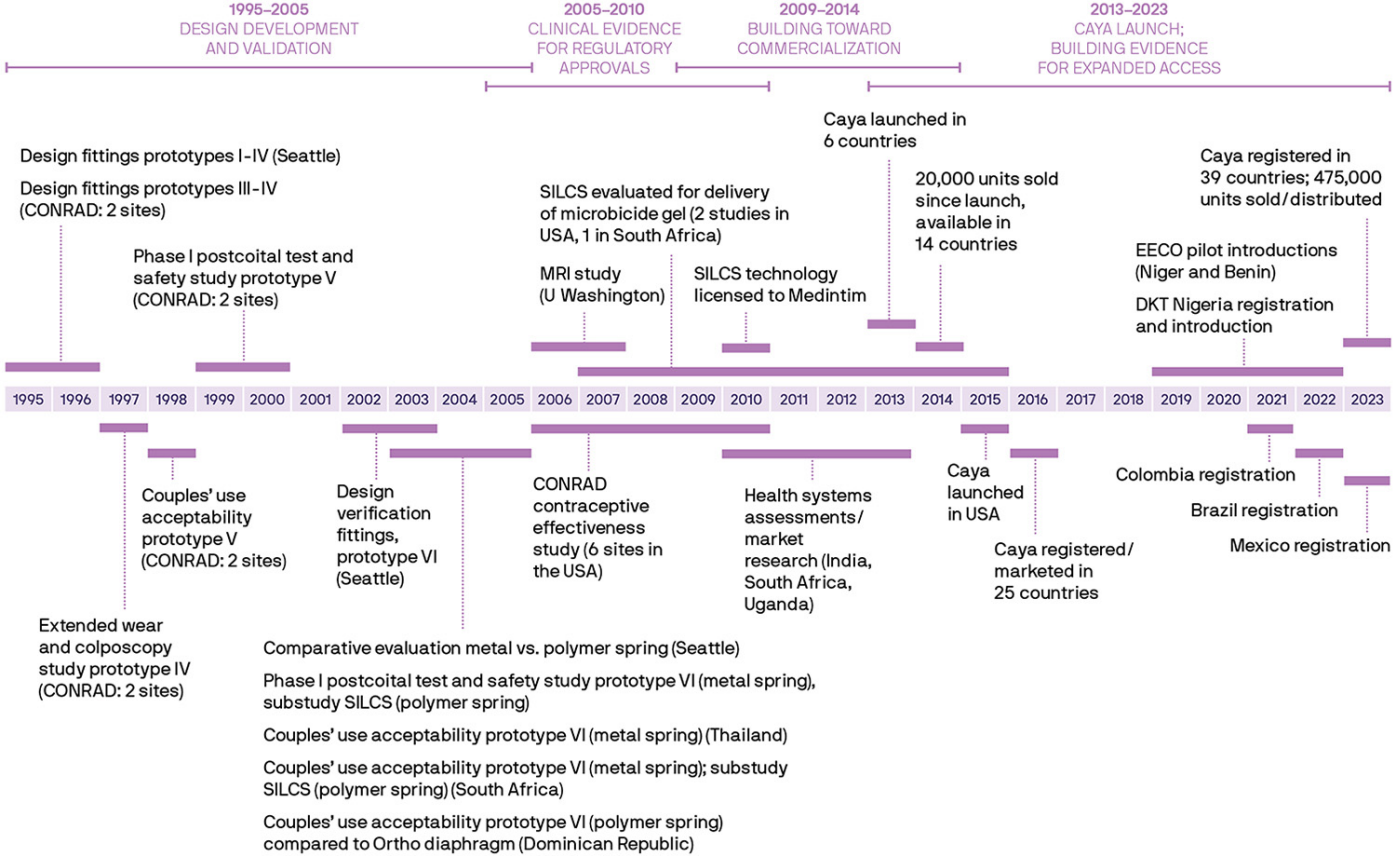
Timeline of Development of Caya Contoured Diaphragm Abbreviations: EECO, Expanding Effective Contraceptive Options; MRI, magnetic resonance imaging; U, university; USA, United States of America.

Prototypes were refined after each evaluation based on user feedback. User/provider feedback helped refine features such as material thickness, shape, and spring design. User insights led to features, such as the grip dimples and removal dome, to aid ease of handling, insertion, and removal. The design evolved through 6 prototype iterations as we sought to balance ease of insertion and comfort with a device that would fit a broad range of women (i.e., in terms of cervical position, fit with traditional diaphragm size, body mass index, and parity). The final diaphragm design (Figure 1) is more anatomically shaped and has a contoured spring (Figure 2), allowing it to fit a wide range of women. It has a softer spring force than traditional diaphragms, grip dimples to aid insertion, a finger dome to aid in removal, and a design that does not impinge on the urethra. Figure 3 shows the timeline of this early development work, in addition to subsequent studies and, ultimately, commercialization and product launch. The single-sized diaphragm was called SILCS during product development and is commercialized as the Caya contoured diaphragm.

User/provider feedback helped refine features such as material thickness, shape, and spring design.

As noted in Table 1, donors and providers wanted a single-sized design that would fit most women, obviating the logistical difficulty of supplying multiple sizes, reducing the clinical burden of a fit exam, and enabling service delivery outside the clinic setting to improve access. In formative research conducted from 1995 to 1998, we found that women wanted a device that was “soft,” small, and discreet so it would be comfortable and easy to insert. A challenge was to make a device that was soft but not so soft it could not be easily inserted and positioned over the cervix. Women wanted a diaphragm that would be comfortable to wear, not interfere with sex, and be easy to remove. Women did not want to consider the diaphragm as a medical device. They wanted to think about it as a “friend who works with my body and keeps me protected.” Women were not attracted to previous diaphragms that had been supplied in colors such as pink, white, or beige. They wanted something “fresh” and “modern.”

Understanding how to create a single-sized device that fits a broad range of women was a key technical challenge. We explored the fit of different styles of diaphragms, cervical caps, and vaginal pessaries to understand fit dynamics. We worked with vaginal anatomy experts to understand vaginal musculature to explore how to create a diaphragm that did not require a wedged fit between the posterior fornix and the pubic bone but rather would “home” over the cervix and, like a cervical cap, keep the cervix covered as the vagina elongates (tents) during sex.

## BUILDING EVIDENCE OF DIAPHRAGM ACCEPTABILITY FOR WOMEN IN LOW- AND MIDDLE-INCOME COUNTRIES

Working with partners in multiple countries, we conducted iterative studies to collect feedback that informed refinement of the design; build evidence about fit, acceptability, and ease of use; and, eventually, to assess barrier protection from sperm (Table 2).^22^^–^^35^ Results from these studies showed women from diverse geographic regions, representing a range of body mass (underweight/normal/obese) and parity (nulliparous to para 7), could fit and use the single-sized diaphragm. The contraceptive effectiveness study implemented by CONRAD and its partners at 6 sites in the United States^22^ confirmed that effectiveness of the single-sized diaphragm was similar to the traditional diaphragm when both were used with contraceptive gel. This study provided additional confirmation that the single-sized diaphragm fits most women who could wear a traditional diaphragm. The single-sized diaphragm fit 98% (439/450) of women in this study. Most women could successfully insert and confirm fit based on the instructions for use, with no coaching or counseling. Studies confirmed the single-sized diaphragm fits women who normally would wear size 65–80 mm traditional diaphragms.

**TABLE 2.** Summary Findings From SILCS Acceptability Studies and Pilot Introduction Activities^a^

**Summary Findings**	**Countries (Year of Publication)**	**Notes**
SILCS diaphragm is easy to use and acceptable in low- and middle-income countries.	Dominican Republic (2006, 2008); South Africa and Thailand (2006, 2008)	Women who had never used a diaphragm before found the SILCS diaphragm easy to use. Both women and men found it to be acceptable.^25^^,^^26^^,^^30^
	Niger (2022, 2020); Benin (2022)	Women in Niger and Benin reported the Caya diaphragm was easy to use and acceptable.^31^^–^^33^
In comparative studies, SILCS diaphragm is preferred over other diaphragms and cervical barriers.	Dominican Republic (2008); United States (2002)^b^	Women and their partners preferred the SILCS diaphragm^26^ compared to the Ortho All-Flex diaphragm.
	Zimbabwe (2010)	Young women preferred SILCS to the Ortho All-Flex and FemCap.^27^
Confidence and ease of use increase with experience.	South Africa (2015, 2018)	Women with no previous diaphragm experience were able to confidently insert the SILCS diaphragm after 2–3 uses.^34^^,^^35^
SILCS diaphragm does not interfere with sensation and pleasure during sex.	Dominican Republic (2006, 2008); South Africa and Thailand (2006, 2008)	Women reported good comfort and sensation in 83%–86% of product uses; men reported good comfort and sensation in 64%–90% of uses.^25^^,^^26^^,^^30^
	South Africa (2015, 2018)	Women reported less gel messiness and leakage during intercourse when gel was delivered using the SILCS compared to an applicator.^34^^,^^35^
Reuse of SILCS diaphragm is acceptable.	Dominican Republic, South Africa, Thailand (2010)	Women had no difficulty washing and reusing the diaphragm; women and their partners found doing so to be unproblematic.^13^
Insertion of SILCS diaphragm is acceptable, even to young women.	Zimbabwe (2010)	Women aged 16–21 years were not discouraged from inserting or removing the device and having to touch their genitalia.^27^
SILCS diaphragm fits most women and is easy to learn to insert and use.	Dominican Republic (2006, 2008); South Africa and Thailand (2006, 2008); South Africa (2015, 2018); United States (2015)	Women, including those with no previous experience with diaphragms, reported high acceptability for ease of use (insertion/removal), comfort and sensation. Good sensation and comfort for male partners.^25^^,^^26^^,^^30^ The SILCS diaphragm fit women representing a range of body mass and parity.^25^^,^^26^^,^^30^^,^^34^ In South Africa, 70% of women reported insertion was easy after just one use; ease of use and comfort improved with additional experience.^34^^,^^35^ The quick learning curve for new users was an important result of the new design. In the contraceptive effectiveness study, 76% of women were able to insert and correctly position the diaphragm on the first attempt after looking at the written and pictorial instructions (no coaching). With coaching, 94% of women were able to insert, correctly position, and remove the diaphragm, per clinician assessment.^22^
Women/couples report high continuation of SILCS/Caya as their contraceptive method.	Niger and Benin (2020; 2022)	Six months after initiation, 76.7% of survey respondents (N=150) in Niger and 76.5% in Benin (N=225) reported continued use of Caya. Top reasons for continued use across all respondents in both countries were that Caya caused no side effects for most users, worked on demand, and was reusable for up to 2 years.^31^^–^^33^

^a^ Adapted from MatCH Research Unit 2016,^42^ with additional data from Expanding Effective Contraceptive Options (EECO) project publications,^31^^–^^33^ and other cited sources.

^b^ CONRAD, 2002, Phase I Comparative Postcoital Testing and Safety Study of the SILCS diaphragm vs. the Ortho All-Flex diaphragm; unpublished.

At the time of the ICPD, few global health practitioners believed that a contraceptive diaphragm was a feasible option for women in LMICs. Despite studies in Colombia, India, the Philippines, and Turkey involving traditional diaphragms reporting relatively high acceptability,^10^^,^^36^^,^^37^ diaphragms were not widely available or promoted. Researchers also called for increased attention to the diaphragm as a contraceptive as well as to potentially protect from sexually transmitted infections (STIs), either alone or as a delivery system for microbicide gel if one became available.^9^^,^^38^

In preparation for introduction of the single-sized diaphragm, research teams in India, South Africa, and Uganda implemented health systems assessments and preliminary market research that explored potential interest in and feasibility of future introduction of the single-sized diaphragm^39^^–^^41^ to begin characterizing factors that would support introduction. They explored the policy and procurement environment, the regulatory pathway, potential service delivery options, and perspectives of women likely to be interested in the SILCS diaphragm. These assessments confirmed interest in the diaphragm as a method that could increase choice, reduce the gap of unmet need for FP, and potentially provide a backup option when other methods are out of stock. But, as a totally new method, it would require education and awareness-raising among providers, other stakeholders, and potential users.^39^^–^^43^ The South Africa research team also compiled a policy landscape indicating the policy environment was conducive to introduction^44^ and a policy brief summarizing evidence from studies and advocating for increased access to SILCS to expand options for nonhormonal contraception and/or as a delivery system for microbicide gel.^42^ Researchers also implemented a cost-effectiveness analysis for Gauteng province in South Africa that evaluated potential costs and health impact for future introduction of SILCS diaphragm as a contraceptive^45^ to inform future discussions. This analysis concluded that public-sector introduction of the SILCS diaphragm could potentially reduce unintended pregnancies, especially if introduction focused on women not currently using existing methods. The evaluation found that from the provider’s perspective, the introduction of the SILCS diaphragm would cost between US$125 and US$171 (in 2011 US$) per pregnancy averted, depending on adherence. Results were especially sensitive to the cost of the contraceptive gel but were also sensitive to the assumptions for a mass media campaign and training costs. The study concluded that depending on the government’s willingness-to-pay threshold, the SILCS diaphragm could add value and contribute to reducing unmet need for contraception in South Africa.

Assessments in LMICs confirmed interest in the diaphragm as a method that could increase choice, reduce the gap of unmet need for FP, and potentially provide a back-up option when other methods are out of stock.

Researchers began to assess diaphragm use for STI protection—either on its own to cover the cervix or as a delivery system for microbicide gel if one became available. Researchers implemented studies of traditional diaphragms in multiple countries, including Kenya, Madagascar, South Africa, and Zimbabwe. Many of these studies reported good acceptability and willingness to use, confirming that even in challenging settings, women can learn to use a diaphragm and use it safely and successfully. A South African study evaluating potential acceptability of the SILCS diaphragm as a microbicide gel delivery system found that more women were interested in SILCS/microbicide gel for multipurpose protection (68%) than in either SILCS alone as a contraceptive (17%) or microbicide gel alone for STI protection (14%).^34^^,^^35^ Unfortunately, after the multicountry trial evaluating use of a traditional diaphragm for HIV protection reported that the diaphragm (used with lubricant gel and male condoms) was no more effective at preventing HIV infection than a condom alone,^46^^,^^47^ and early microbicide gel products failed to show efficacy against HIV in clinical trials,^48^^,^^49^ interest in diaphragms for STI protection diminished as HIV prevention strategies moved to longer-term drug delivery systems and more potent drug candidates.

Some microbicide gel products are still being developed as multipurpose prevention technologies (MPTs) aimed at protecting from STIs and unintended pregnancy. For example, Yaso-GEL^50^ is an MPT gel being developed to protect from Neisseria gonorrhoeae, herpes simplex virus type 2, and pregnancy. The U.S. Food and Drug Administration recently approved Yaso-GEL’s Investigational New Drug application for a first-in-human study (Phase 1). If an MPT gel like this becomes available in the future, it could be used in conjunction with the Caya diaphragm to enhance protection from unintended pregnancy and STIs among women seeking to use nonhormonal contraception.

## STRENGTHENING UNIVERSAL ACCESS TO SEXUAL AND REPRODUCTIVE HEALTH CARE

In 2010, PATH licensed the single-sized diaphragm technology for commercialization to Medintim GmbH (Walldorf, Germany), a family-operated company that manufactures and distributes sexual and reproductive health products through a network of country partners.

An advantage of partnering with Medintim was that they already sold a lactic acid-based contraceptive gel product that had a long history of acceptability and use with diaphragms and cervical caps and has Conformité Européenne regulatory approval for the European Union and approval in many other countries. The International Planned Parenthood Federation in London developed the formulation in 1972 to provide an alternative gel option for women who disliked spermicide gel containing nonoxynol-9. The Dutch Family Planning Association produced this gel for diaphragms as Contracep Green from 1980 to 2003; they then released the formulation to Medintim, who has marketed it as Contragel since 2005 (personal communication, July 12, 2024, with Medintim).

After technology transfer, production scale-up, and regulatory submissions, the Caya contoured diaphragm became a reality, with product launch in 2013.^51^ At that time, Medintim launched a rebranded version of the lactic acid-based gel as Caya Gel^52^ to be used with the Caya diaphragm. CONRAD subsequently implemented 2 studies of Medintim’s Contragel (Caya Gel) to explore safety and effectiveness when used with the diaphragm. The first study found that the Caya diaphragm used with Contragel was safe and performed as well as the Caya diaphragm used with nonoxynol-9 spermicide at providing a barrier to forwardly motile sperm.^53^ The second study found no clinically significant differences between the safety profiles of the lactic acid diaphragm gel versus hydroxyethylcellulose placebo gel when used with the study diaphragm.^54^ These studies provided evidence that Caya Gel is safe for use even in countries with high risk of HIV.

The Caya diaphragm was initially launched in 6 countries and now is approved and registered in nearly 40 countries. More than 475,000 units had been sold or distributed as of January 2024, a result of the 30 years of effort since ICPD. Because the Caya diaphragm is not yet included in procurement by international donors, access was achieved by introducing the product through Medintim’s network of distribution partners in countries where partners believed in the product and were interested in expanding access to a nonhormonal, on-demand method for women. Many of these partners are small and family-operated companies or female entrepreneurs committed to supplying sexual and reproductive health products to their communities. They understand the product, believe there is a role for expanded access to a nonhormonal, user-initiated method, and are working to reach consumers who might be interested in this method. The success of their efforts points toward the opportunity to expand access to Caya in middle-income and higher low-income markets where women access FP through private-sector channels. The Expanding Effective Contraceptive Options (EECO) project also implemented 2 public-sector investments exploring Caya diaphragm pilot introductions in Benin and Niger (2019 to 2022). These short-term pilot introductions explored uptake and acceptability of the Caya diaphragm and Caya gel through public- and private-sector channels and found high levels of acceptability, with more than 76% of respondents reporting continued use at 6 months.^31^^–^^33^

Short-term pilot introductions in Niger and Benin explored uptake and acceptability of the Caya diaphragm and Caya gel found more than 76% of respondents reporting continued use at 6 months.

Based on the positive results of these pilot introductions in Niger and Benin, the U.S. Agency for International Development (USAID) invited Medintim in 2021 to apply for the Caya diaphragm and Caya Gel to be included in the USAID procurement manual, which could facilitate public-sector availability in countries that USAID supports. The Caya diaphragm has been recommended for inclusion in the procurement manual, but the Caya Gel was not recommended due to concerns about its 2-year shelf life, temperature storage requirements, and the recurring cost of the gel. In response to this feedback, Medintim is reformulating the product to achieve a 36-month shelf life and storage stability in climate zone IVb (30°C; 75% relative humidity). They also are planning to transfer production to a more automated facility to reduce gel manufacturing costs.

Regulatory approvals and clinical guidance recommend diaphragms be used in conjunction with a contraceptive gel (formerly defined as a spermicide) to improve effectiveness. The Caya contraceptive efficacy study that provided clinical data for regulatory approval evaluated the Caya diaphragm used with a contraceptive gel.^22^ Because no appropriate contraceptive gel that meets public-sector procurement specifications is available, the Caya diaphragm is not yet available through public-sector procurement. Researchers and providers have questioned the additive value of the contraceptive gel to the physical barrier provided by the diaphragm. A Cochrane review in 2003 evaluating the efficacy of diaphragm with and without spermicide reported insufficient clinical evidence and recommended additional studies to answer this question.^55^ Also, little is known about how diaphragm users use gel in real life. Caya Gel comes in a 60-ml, multidose tube (estimated at 15 doses per tube), but little is known about how much gel women use or if they use gel each time they use the Caya diaphragm. Even in the contraceptive efficacy study in which women were counseled to use the Caya diaphragm and contraceptive gel together, women reported not using the gel in about 15% of coital acts.^22^ While it makes logical sense that a diaphragm used with contraceptive gel would be more effective than a diaphragm alone, the CONRAD postcoital testing study, which compared barrier effectiveness of the Caya diaphragm used with a lactic acid-based contraceptive gel to Caya diaphragm used with nonoxynol-9 spermicide and Caya diaphragm used with no gel, found that the Caya diaphragm with no gel was nearly as effective at stopping forwardly motile sperm as Caya used with either Caya Gel or nonoxynol-9 spermicide. The results were not statistically different between the 3 arms of the study.^53^ This has led some researchers and program planners to question the need to supply diaphragm and contraceptive gel together. However, the regulatory approvals recommend use of the diaphragm with gel. Also, results from the pilot introductions in Niger and Benin found that women liked the gel, perceived it as something special, and felt that it made sex pleasurable. Because Caya is not yet available through public-sector procurement and programming, Medintim has built access through private-sector and social marketing groups that are able to import and sell both the Caya diaphragm and Caya Gel.

## RESEARCH AND CONSUMER EXPERIENCE IS HELPING BREAK DOWN MISCONCEPTIONS

Caya diaphragm introduction and consumer experience, as well as research with traditional diaphragms, is helping break down misconceptions about diaphragms (Table 2). This is changing the narrative about diaphragms and brings new light to understanding consumers who want to use a nonhormonal barrier method. Previously, diaphragms were sidelined as a method appropriate only for older women. However, recent pilot introductions of the Caya contoured diaphragm in Benin and Niger aimed at generating evidence to build support for public-sector introduction have shown this is not the case and found high rates of acceptability and continued use in both countries. Because Caya is discreet, on-demand, and does not impact bleeding patterns or sexual pleasure, it has the potential to address the sexual and reproductive health needs of young women^56^^,^^57^ Because it is reusable for at least 2 years, it can reduce waste seen with other short-acting methods. The Caya diaphragm was designed to be easy to use, especially for new users. The quick learning curve for new users is an important aspect of reducing training costs and expanding the market. Caya can provide a backup method of contraception when other methods are stocked out and can also be used in conjunction with other methods, such as natural FP, to provide protection during the fertile period when couples are advised to avoid unprotected sex or abstain.

Consumer experience and research is changing the narrative about diaphragms and brings new light to understanding consumers who want to use a nonhormonal barrier method.

Women representing a range of ages, marital and relationship status, parity, education, and economic status can use diaphragms successfully. Studies looking at diaphragm use for STI protection indicated that women across a range of ages found diaphragm use acceptable.^46^^,^^58^^–^^65^ Preliminary research in Zimbabwe exploring acceptability of 3 vaginal barrier devices among young women reported that they could insert the devices and found them acceptable.^27^ Results from the annual consumer survey implemented by Medintim among women in Germany found that Caya users range in age from 18 to 40+ years and represented married and unmarried persons with and without children. (Germany is the only country from which consumer data have been collected; results can be accessed via https://www.medintim.de/en/products/contraception/caya-contoured-diaphragm/).

Although some stakeholders have questioned whether women in so-called “low-resource settings” could use the diaphragm successfully, couples’ use acceptability studies in the Dominican Republic, Thailand, and South Africa,^13^^,^^25^^,^^26^ as well as the pilot introductions in Benin and Niger,^31^^,^^33^ showed that couples find Caya acceptable, and women could wash and care for the diaphragm even in those settings. The pilot introductions in Benin and Niger showed the Caya diaphragm to be acceptable even among women who had no previous diaphragm experience. In Niger, almost 77% of respondents to an end-line survey reported continued use of Caya after 6 months; top reasons for continued use included that Caya caused no side effects for most users, worked on demand, and was reusable for up to 2 years.^31^

Some stakeholders questioned diaphragm acceptability because it is recommended for use with a contraceptive gel, and there is a perception this is messy and problematic or would be unacceptable in communities or among couples where “dry sex” is preferred. However, the EECO project team found that some women reported liking the feel of the gel and considered it “precious.” Rather than being a problem, it could enhance sexual pleasure.

Some stakeholders believed Caya would primarily be of interest to women with previous diaphragm experience. Data from early introduction indicates that this is not the case. Data from the Medintim survey implemented over 9 years shows that most women have never used a diaphragm before. The women in the Niger pilot had not used a diaphragm before, and approximately half of the women participating in end-of-pilot interviews reported not having used a contraceptive method previously.^31^ These findings suggest that Caya may provide an entry point for new users.

The Caya diaphragm could benefit women in any country where there is unmet need for contraception, existing methods do not meet women’s needs, and/or a nonhormonal, user-initiated method is desired, especially if there are no strong cultural barriers against vaginal methods. Countries in which there is interest in and acceptability of menstrual cups may also be early adopters of a method like Caya. For public-sector introduction, prioritizing countries with low contraceptive prevalence, limited acceptability of hormonal methods, or frequent stock-out of other short-acting methods might bring the greatest benefit. For private-sector introduction, countries with strong private-sector markets, where women already access contraceptives through private-sector channels and consumers are not overly reliant on the public sector for contraceptives, might provide the best opportunities for building a market.

## PROGRAMMATIC LESSONS FOR INCREASING UNIVERSAL ACCESS

Experiences with early introduction of the Caya diaphragm provide a new model for increasing access to novel and underutilized methods, especially through private-sector partners. Introduction across multiple countries provides insight into strategies country partners have used to raise awareness and expand access to this new method. The strategies have differed by country context, the strength and expertise of the country partner, and their ability to access potential channels and clients. Most supply to a range of channels, including clinics, pharmacies, and providers, as well as online marketing. Following this innovative model, Caya is now registered and being marketed in nearly 40 countries.

Experiences with early introduction of the Caya diaphragm provide a new model for increasing access to novel and underutilized methods.

The Caya diaphragm is uniquely positioned to address the demand for nonhormonal, on-demand contraception. Pilot introductions show that the initial concerns from providers and program implementers did not materialize—women are willing to touch themselves to insert this product and can indeed take care of it. A 2023 market dynamics assessment identified Caya as a “very niche product” that will require “resource-intensive demand generation,” and results from modeling suggested that most Caya adopters would be “switchers” rather than “new users” of contraception (unpublished data). However, the report also acknowledges that little is known about market size, market segmentation, or willingness to pay because introduction has been limited and these investments have not been made.

The following lessons and strategies could help inform future introduction of other over-the-counter methods still in development.

### Find the Right Kinds of Partners

Key to the Caya diaphragm introduction has been having country partners who not only know how to register and import a medical device but also understand the Caya diaphragm as a method and believe there is a role for expanding access to a nonhormonal user-initiated option for women. These partners are committed to providing sexual and reproductive health products and services to their communities. They build relationships, bringing this new method to women in ways that meet their needs, and do not view introduction as a short-term project.

### Find Right-Sized Partners

Partners who have been successful at Caya diaphragm introduction have been not too small or too large. The Caya diaphragm and gel should be part of a portfolio of products. If the Caya diaphragm is the only product they sell, there is too much risk for the partner if the product is not successful. If the partner portfolio is too large, the Caya diaphragm will not be prioritized as an important part of their business.

### Build a Sustainable Supply Chain

The supply chain is not just focused on ensuring supply of the diaphragm and gel but must also include an educational component for women, providers, and other stakeholders. This can be scaled according to available resources to deliver key messages in a way that meets users’ needs.

### Raise Awareness About the Method

The Caya diaphragm is a new method, and it takes time to raise awareness. Even in countries where diaphragms were available in past decades, providers and other stakeholders have limited knowledge about this method.

### Reach Out to Sexual and Reproductive Health Providers With Education and Training

Even in countries where the Caya diaphragm is available over the counter, it is important to reach out to providers with materials and training so that they have accurate information. Provider attitudes can be an obstacle to accessing the diaphragm partly due to lack of familiarity with the method and concerns about its use.^66^

### Share Information With Women About How to Use Caya Safely and Correctly

The Caya diaphragm is not difficult to use, but it requires basic understanding of the vagina, the cervix, and the pubic bone to learn how to insert and remove the device. Women need information from a trusted source to decide if this is a good method for them and learn how to use it safely and correctly. This source may be a community health worker or peer group, as long as it is someone who understands and can speak authentically about the method. Some partners have creatively leveraged networks in which women share information on sensitive topics, for example, combining the Caya diaphragm introduction with menstrual hygiene education and in support groups for fertility awareness and sexual health trainings.

### Allow Time for Unformed Markets to Grow

As a new method, it takes more than getting the Caya diaphragm into the pharmacy or clinic for women to accept this method. Partners have started with small initiatives and limited distribution and have explored how to reach women and share information and products in a way that works for the client.

### Invest in Awareness-Raising and Education

Diaphragms have not benefited from the public-sector investments in recent decades that have supported training, access, and availability of other FP methods, so partners need to invest in awareness-raising and education to create a supportive environment for introduction. Partners who are growing the market in their country include an education component that reaches women through channels that resonate with their audience. They educate not just about product use but also about sexual and reproductive health and menstrual health—information that all women benefit from.

Partners need to invest in awareness-raising and education to create a supportive environment for introduction.

### Explore Different Distribution Models for Different Geographies

The Caya diaphragm was launched first through private-sector partners in high-income countries where country partners expressed interest. Because it is not included in public-sector procurement and some stakeholders had questions about whether women in LMICs could use a diaphragm, this seemed a good way to explore uptake and acceptability. Early success through private-sector channels led the social marketing groups at DKT Nigeria and DKT Brazil to explore introduction. DKT introduced the Caya diaphragm through its distribution channels to pharmacies, clinics, and online shops. Unfortunately, both partners found it difficult for the Caya diaphragm to price compete in markets where other contraceptive methods were better known, already integrated into the health system, and highly subsidized. This is leading them to reassess whether Caya fits within their portfolio at this time.

### Be Innovative About Introduction

Because the Caya diaphragm is not intended to compete with other contraceptive methods, such as hormonal methods and intrauterine devices, this encourages partners to be creative in reaching women who may not currently be visiting FP clinics. Because the Caya diaphragm can be provided outside clinic settings, this provides opportunities for innovative ways to reach potential consumers through grassroots and peer-to-peer communication, using existing networks in which women share sensitive information.

### Be Creative in Terms of Distribution Partners and Platforms

To date, Caya diaphragm country partners often are small, woman-run start-ups or family-operated small businesses that are willing to start small and invest in the time it takes to make a real connection with potential clients. Because they have limited capital to invest, they have been creative at identifying economical ways to reach potential clients through existing networks and communication channels. For example, in Colombia, a social media influencer who has an online shop for sexual health products has created videos about the Caya diaphragm to raise awareness and generate sales. In Hungary, the country partner uses her workshops in sexuality education to raise awareness among women who are interested in natural FP. In Israel, the country partner supplies the Caya diaphragm through clinics and an online shop aimed at addressing the sexual health needs of women, including women from conservative religious communities.

### Create Opportunities for Real-World Assessment of Willingness to Pay to Better Understand Market Opportunities in Low- and Middle-Income Countries

The pilot introductions in Benin and Niger provided some data about willingness to pay but are not realistic data points unless the product is subsidized. For example, in Niger, the Caya diaphragm and Caya gel were either free if accessed through the public sector or highly subsidized when accessed through community health workers or private clinics (500 CFA [US$0.90] for a diaphragm and gel kit and 500 CFA [US$0.90] for resupply of the Caya gel [estimated 15 uses per tube]). Benin also alternated between subsidized sales and free distribution. The price was subsidized because the primary objective of these pilot introductions was to assess uptake and acceptability of this new method. Exploring willingness to pay and service delivery preferences were secondary objectives. The DKT Nigeria introduction (2020 to 2022) supplied the Caya diaphragm through multiple channels, including pharmacies, clinics, and online shops, at a price that reflected all costs. After accounting for margins in these various channels, the consumer prices ranged from 10,000 to 15,000 Naira (US$24 to US$36) (2021).^67^ This was at the high end of willingness to pay for contraception in Nigeria, especially for a new method with limited awareness. Only high-income consumers could afford Caya at this price point. The majority of women for whom Caya might be a great fit did not know about the product and could not afford this fully loaded retail cost.

### Develop Sustainable Pricing for the Unsubsidized Market

The Medintim unit price for Caya diaphragm and Caya gel to LMICs is around US$5.66 and US$3.76 per unit, respectively. More information is needed to better understand how to support private-sector and social marketing-sector introduction in LMICs, to explore willingness to pay and to help partners access feasibility for sustainable markets, especially in markets where other contraceptives are subsidized. In the meantime, Medintim is working to reduce landing costs by decreasing the cost of the Caya Gel and exploring alternative and efficient transportation for small-volume orders.

## A LONG ROAD FROM THE INTERNATIONAL CONFERENCE ON POPULATION AND DEVELOPMENT TO PRESENT

At the time of the 1994 ICPD, women’s health advocates identified women’s desire for more methods they could control, have few side effects, and could protect from unintended pregnancy and potentially STIs. Recent evaluations of reasons for unmet need confirm that concerns about health consequences, side effects, effects on libido, and wanting a method that can be used when needed still exist.^56^^,^^68^^,^^69^ Diaphragms are already included in the World Health Organization List of Essential Medicines, which guides country-level selection and financing and can influence support for universal health coverage and access to essential medicines.^70^^,^^71^ The recent revisions to the analysis of couple-years of protection (CYP)^72^ included diaphragm among the contraceptive methods listed (after several years of not being included), signaling that diaphragms could become more widely available in the future. CYP is an indicator used by international organizations and country governments to measure FP program outputs and make assumptions about estimated contraceptive protection based on the volume of methods sold or distributed during 1 year. CYP informs forecasting assumptions for public-sector procurement ordering and can influence prioritization of program investments for introduction, education/training, and demand creation.

It takes time and investment to introduce a new method and build sustainable markets. It is important to strategically consider where this method can have a likelihood of success and help address women’s needs and how to reach consumers who are interested in this new and underutilized option. The demands voiced by women 30 years ago at ICPD have not yet been fully addressed; the Caya contoured diaphragm is poised to help fill that gap by expanding universal access to a method of FP that meets their needs.

## Notes

**Peer Reviewed**

**First Published Online:** October 3, 2024.
